# Postsurgical pyoderma gangrenosum and flap necrosis in a head and neck cancer patient following neck dissection

**DOI:** 10.1002/ccr3.2828

**Published:** 2020-04-08

**Authors:** Julia Arebro, Björn Palmgren

**Affiliations:** ^1^ Division of Otorhinolaryngology Department of Clinical Science, Intervention and Technology Karolinska Institutet Stockholm Sweden; ^2^ Department of Otorhinolaryngology, Head and Neck Surgery Karolinska University Hospital Stockholm Sweden

**Keywords:** flap necrosis, head and neck cancer, neck dissection, postsurgical pyoderma gangrenosum, pyoderma gangrenosum

## Abstract

Postsurgical pyoderma gangrenosum (PSPG) develops in the skin after surgery without known cause. Immunosuppression constitutes first‐line therapy and increases the likelihood of successful surgery when needed. PSPG should be considered when a flap necrosis occurs.

## Graphical abstract

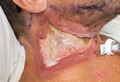

## 1 INTRODUCTION

Pyoderma gangrenosum (PG) is an uncommon dermatosis that can be difficult to diagnose and treat. Postsurgical PG represents a condition where patients develop PG‐like lesions in the skin after surgery. We here demonstrate a case that developed PSPG after surgery for malignancies in the head and neck region.

Pyoderma gangrenosum (PG) is an uncommon dermatosis that can be difficult both to diagnose and to treat. The ulcerative cutaneous lesion is often clinically diagnosed after other causes have been excluded, including infection, malignancy, vasculitis, collagen‐associated vascular diseases, trauma, and diabetes‐associated ulcer. PG most often starts with an initial lesion, like a bite reaction, developing to a papule or pustule and eventually expanding to an ulcerative lesion. A biopsy of the wound edge demonstrating a neutrophilic infiltrate is the single major criterion to help establish the diagnosis.^1^ Up to 75% of the patients have an associated systemic disease, most often inflammatory bowel disease (IBD), arthritis, or hematological disorders.^2^ Some patients demonstrate pathergy which is the development of PG‐like lesions at the site of skin trauma after surgery.

## 2 CASE REPORT

A 58‐year‐old man with liver cirrhosis and ulcer of the stomach in his medical history was found to have a T3N1M0 squamous cell carcinoma in the floor of the mouth between teeth 33‐43. The tumor was surgically resected, and the patient was reconstructed with a free anterior lateral thigh (ALT) flap. At the same procedure, a neck dissection region 1‐5 right side and region 1 left side was carried through since fine needle aspiration cytology had confirmed a regional metastasis in region 1, right side. The patient received antibiotics the same day as the surgery and regularly the days after surgery. However, the patient developed a redness on the neck and on the flap donor site 5 days after surgery, and pus started to leak from the neck wound.

**FIGURE 1:**
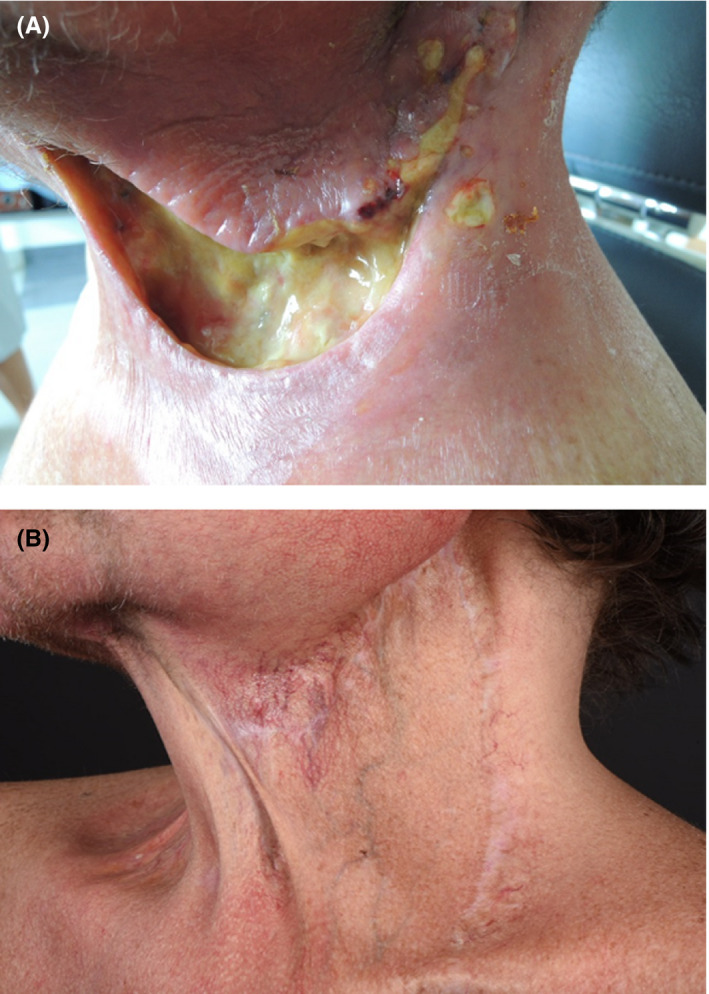
Left neck 2 wk after neck dissection (A) with manifestation of pyoderma gangrenosum and (B) healed

Three years previously, the patient had been diagnosed with left‐sided tonsil cancer and ipsilateral metastases on the neck. He then received full‐dose (68Gy) radiotherapy. At the 3 months follow‐up, a CT scan was routinely done and showed remaining lymph nodes with suspicious appearance. A neck dissection of the ipsilateral side regions 1‐5 was carried out. Three days after surgery, a redness and suspected infection on the neck was found and clindamycin was ordinated. The patient was dismissed from the hospital but returned 13 days after surgery since the neck did not improve (Figure 1A). The neck showed alarming redness and the surgical wound was purulent. The patient was put into the ward and ordinated intravenous cefotaxime and later, cloxacillin, since a swab showed *s aureus*. Despite adequate antibiotics, a flap necrosis developed, and the neck was closed a month after the neck dissection with a transposition flap. The flap area got red after surgery, and a local swab showed presence of *enterococcus* and *coagulase‐negative staphylococcus*. The patient was treated with several courses of antibiotics, and the flap eventually healed without necrosis about a month after the flap surgery (Figure 1B).

**FIGURE 2:**
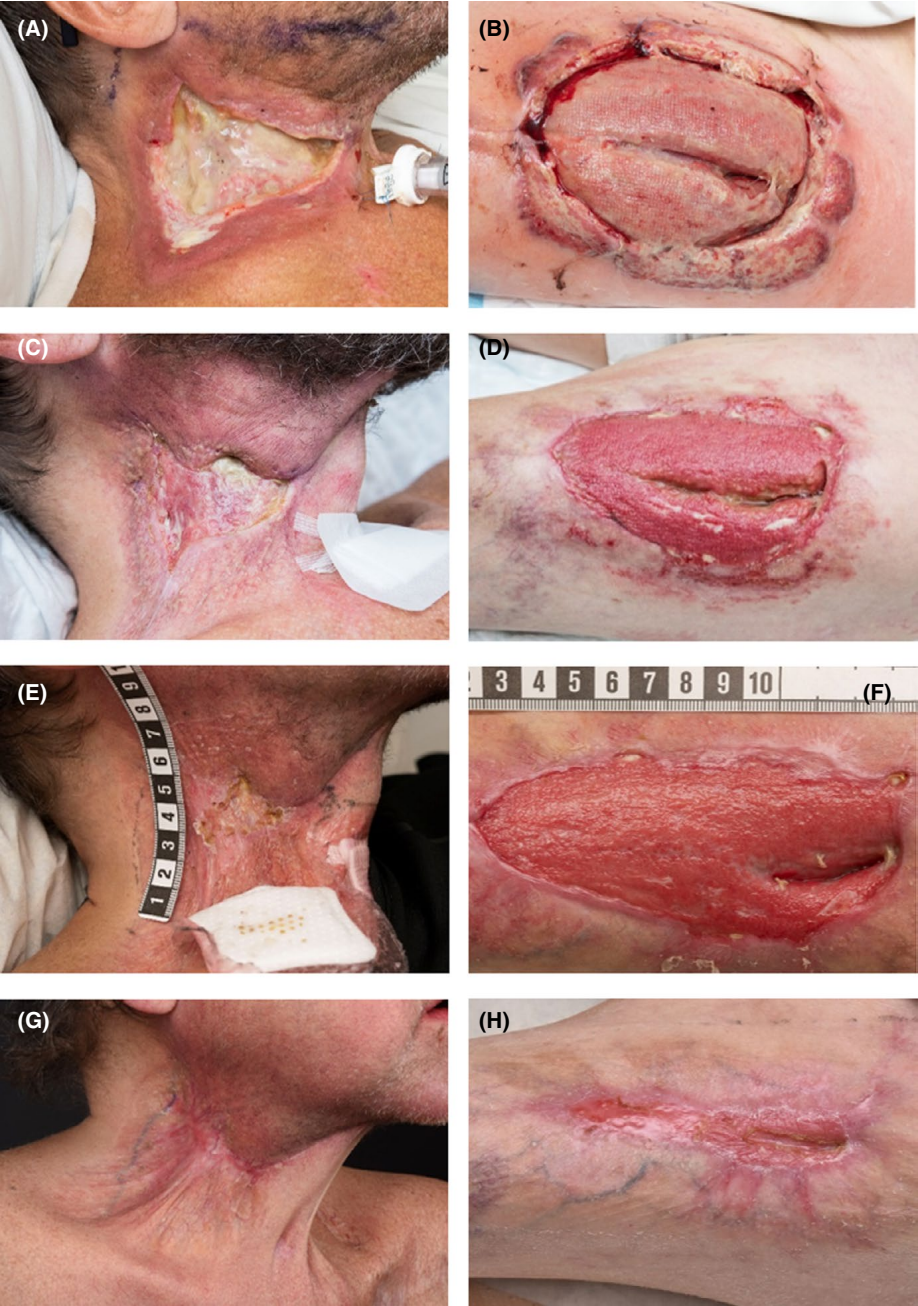
Right neck and left thigh with manifestation of pyoderma gangrenosum 2 wk (A and B), 5 wk (C and D), 2 mo (E and F), and 4 mo (G and H) after primary surgery

After the recent surgery for the cancer in the floor of the mouth, the flap of the neck eventually developed necrosis. The donor site on the thigh ruptured and started to show signs of a superficial infection and the edges developed necrosis, exposing the muscles underneath (Figure 2A,B). Treatment with antibiotics continued although no local swabs showed presence of any bacteria. Thirteen days after surgery, the diagnosis PG was suggested. A dermatologist confirmed the diagnosis clinically, and biopsies from the thigh were congruent with the diagnosis PG. The biopsies showed no bacterial structures but intradermal inflammation with abscess reaching to the deep dermis. The patient begun treatment with oral prednisolone 40 mg daily. The wounds were taken care of daily, and the thigh, concluded to be the most affected area, received vacuum‐assisted closure therapy. The patient got gradually better and 21 days after surgery, prednisolone was reduced to 35 mg daily (Figure 2C,D). One and a half month after the primary surgery, the wound on the leg was covered with a partial skin graft. However, the graft had difficulties to attach and it was eventually rejected (Figure 2E,F). The neck and the thigh healed secondary at last, about 2 and 4 months after surgery, respectively (Figure 2G, H). Four months after primary surgery, the dose prednisolone was reduced by 5 mg every other week.

## 3 DISCUSSION

Postsurgical pyoderma gangrenosum (PSPG) represents a condition difficult to treat and even to diagnose. Recent reviews confirm that comorbidities are rarer in PSPG compared to in classic PG.^3^, ^4^ In PSPG, the cutaneous ulcer often expands rapidly, typically beginning between 4 days and 6 weeks after surgery,^4^, ^5^ with potentially devastating consequences. A rapid diagnosis is therefore necessary to minimize complications. The condition mimics a wound infection with fever, wound redness and purulence and is commonly treated with antibiotics and debridement initially. However, adequate antibiotics do not heal the lesions and unfortunately, debridement is detrimental in PG since it aggravates the pathergy.^6^, ^7^, ^8^ Swabs should be taken from the ulcer to differentiate PG from an infection. Somewhat confusing, however, PG wounds rather often show positive cultures.^9^ The most common areas of PSPG is the breast followed by abdomen/thorax and lower extremities. It is far more uncommon in the head and neck area (3%‐5%), why it often is mistaken for a wound infection to begin with.^3^, ^4^

Pyoderma gangrenosum is thought to be an immunological response to injury and neutrophil dysfunction. IL‐8 has been shown to be overexpressed in PG ulcers. Recombinant human IL‐8 induced similar ulcers in human skin xenografts.^10^ Others have found an overexpression of IL‐1b, TNF‐a, IL‐8, IL‐17, and metalloproteinase (MMP) 9 in PG lesions.^11^, ^12^ A histopathological diagnosis is however difficult, since ulcerations with neutrophilic infiltration are seen in several other conditions. The fact that PG often is a “diagnose of exclusion” has been criticized and in 2018, a consensus was published suggesting one major and eight minor criteria in diagnosing the condition.^1^ In addition to the major criteria, a biopsy of the ulcer demonstrating a neutrophil infiltrate, the patient must have at least four minor criteria to meet diagnostic criteria (Table 1). The authors concluded that this yielded a sensitivity of 86% and a specificity of 90%.^1^

**TABLE 1:** Diagnostic criteria for classic ulcerative pyoderma gangrenosum according to Maverakis et al^1^

Major criteria	A biopsy of the ulcer demonstrating a neutrophil infiltrate
Minor criteria	Exclusion of infection
	Pathergy
	History of IBD or inflammatory arthritis
	History of papule, pustule, or vesicle ulcerating within 4 days of appearing
	Peripheral erythema, undermining border, and tenderness at ulcerating site
	Multiple ulcerations, at least one on an anterior lower leg
	Cribriform or “wrinkled paper” scar(s)
	Decreasing ulcer size within 1 mo of initiating immunosuppressive medication(s)

Note

In addition to a biopsy demonstrating a neutrophilic infiltrate, patients must have at least four minor criteria to meet diagnostic criteria.

There is a lack of complete clinical guidelines addressing the management of PG. In one of few randomized controlled trial, 121 patients were treated with either prednisolone or cyclosporine.^13^ The treatments had similar efficacy, with 15%‐20% of the ulcers completely healed in 6 weeks and 47% at 6 months. Another randomized controlled trial^14^ proved infliximab to be a suitable treatment for PG. However, corticosteroids and cyclosporine are considered the first‐line therapy for PG.^2^, ^15^, ^16^, ^17^ Regardless of early dermatology consultation and correct diagnosis, the aesthetic outcomes can be disastrous, with considerable cribriform scarring (Figure 1B; Figure 2G, H).

Some authors claim surgery to be required in PG^18^ whereas others^6^, ^7^, ^8^, ^19^ regard it as contradicted. There is currently a paucity of data on approach to the patient with PG requiring surgical intervention. If surgery is needed, the recommendation is to have the patient on immunosuppressive therapy.^3^, ^20^, ^21^

## 4 CONCLUSION

Pyoderma gangrenosum is a condition difficult to both diagnose and treat. PSPG refers to the development of PG at surgical wounds in the immediate postoperative period. The clinical signs of PSPG with local redness and purulence mimic a wound infection but antibiotics and wound debridement fail to arrest rapid ulcer enlargement. Immunosuppression is considered the first‐line therapy. The diagnosis PSPG must not be forgotten in cases with flap necrosis in head and neck cancer patients.

## CONFLICT OF INTEREST

The authors declare no conflicts of interest.

## AUTHOR CONTRIBUTIONS

JA: drafted the manuscript, edited photographs, and obtained informed consent from the patient. BP: edited manuscript and contributed to patient care.
